# Correction: A decline in tuberculosis diagnosis, treatment initiation and success during the COVID-19 pandemic, using routine health data in Cape Town, South Africa

**DOI:** 10.1371/journal.pone.0327683

**Published:** 2025-07-03

**Authors:** Karen Jennings, Martina Lembani, Anneke C. Hesseling, Nyameka Mbula, Erika Mohr-Holland, Vanessa Mudaly, Mariette Smith, Muhammad Osman, Sue-Ann Meehan

In the Conclusion subsection of the Abstract, there is an error in the second sentence. The correct sentence is: There was a substantial reduction in the number of individuals diagnosed with drug-susceptible tuberculosis.
