# Supplementary material for: A decline in tuberculosis diagnosis, treatment initiation and success during the COVID-19 pandemic, using routine health data in Cape Town, South Africa
Source: PLoS One. 2024 Sep 11;19(9):e0310383. doi: 10.1371/journal.pone.0310383 (PMC11389921; doi:10.1371/journal.pone.0310383)
Supplement: S1 Table — COVID-19, Coronavirus disease of 2019; DS-TB, Drug- susceptible tuberculosis; HIV, Human immunodeficiency virus; ILTFU, Initial loss to follow-up; PTL, Post-treatment loss. (DOCX) [file pone.0310383.s001.docx]

**Table S1. DS-TB diagnosed, treated, and treatment success in the during-COVID-19 period (April 2020 to March 2021), in Cape Town, South Africa, disaggregated by demographic and clinical characteristics.**

|  | | **Diagnosed with TB** | **ILTF n (%)** | **Notified & Treated** | **PTL n (%)** | **Treatment success** | **Cascade success** |
| --- | --- | --- | --- | --- | --- | --- | --- |
| **Total** | | 19 800 | 3005 (15.2%) | 16 795 | 4384 (26.1%) | 12 411 | 62.7% |
| **Sex** | Female | 8 445 | 1309 (15.5%) | 7 136 | 1806 (25.3%) | 5 330 | 63.1% |
|  | Male | 11 310 | 1690 (14.9%) | 9 620 | 2568 (26.7% | 7 052 | 62.4% |
| **Age** | Child | 1 663 | 372 (22.4%) | 1 291 | 250 (19.4% | 1 041 | 62.6% |
|  | Adult | 18 131 | 2633 (14.5%) | 15 498 | 4132 (26.7%) | 11 366 | 62.7% |
| **HIV status** | HIV negative | 8 678 | 701 (8.1%) | 7 977 | 1894 (23.7%) | 6 083 | 70.1% |
|  | HIV positive | 8 509 | 1493 (17.5% | 7 016 | 2091 (29.8%) | 4 925 | 57.9% |
| **Category of TB** | New | 14 946 | 2268 (15.2%) | 12 678 | 3159 (24.9%) | 9 519 | 63.7% |
|  | Recurrent | 4 854 | 737 (15.2%) | 4 117 | 1225 (29.8%) | 2 892 | 59.6% |
| **Mode of diagnosis** | Bacteriological | 14 503 | 2127 (14.7%) | 12 376 | 3336 (27.0%) | 9 040 | 62.3% |
|  | Clinical | 5 297 | 878 (16.6%) | 4 419 | 1048 (23.7%) | 3 371 | 63.6% |

COVID-19, Coronavirus disease of 2019; DS-TB, Drug- susceptible tuberculosis; HIV, Human immunodeficiency virus;

ILTFU, Initial loss to follow-up; PTL, Post-treatment loss.
