# Supplementary material for: A decline in tuberculosis diagnosis, treatment initiation and success during the COVID-19 pandemic, using routine health data in Cape Town, South Africa
Source: PLoS One. 2024 Sep 11;19(9):e0310383. doi: 10.1371/journal.pone.0310383 (PMC11389921; doi:10.1371/journal.pone.0310383)
Supplement: S2 Table — * p < 0.05 in bold font; COVID-19, Coronavirus disease of 2019; DS-TB, Drug- susceptible tuberculosis; HIV, Human immunodeficiency virus; ILTFU, Initial loss to follow-up; PTL, Post-treatment loss; TB, Tuberculosis. (DOCX) [file pone.0310383.s002.docx]

Table S2. Comparing initial loss to follow up (ILTFU), post-treatment loss (PTL) and cascade success for all individuals diagnosed with DS-TB in the during-COVID-19 period (April 2020 to March 2021) in Cape Town, South Africa, disaggregated by demographic and clinical characteristics.

| **Variable** | | **ILTFU** | **p value*** | **PTL** | **p value*** | **Cascade success** | **p value*** |
| --- | --- | --- | --- | --- | --- | --- | --- |
| **Total** | | 15.2% |  | 26.1% |  | 62.7% |  |
| **Sex** | Female | 15.5% | 0.280 | 25.3% | **0.043** | 63.1% | 0.273 |
|  | Male | 14.9% |  | 26.7% |  | 62.4% |  |
| **Age** | Child | 22.4% | **<0.001** | 19.4% | **<0.001** | 62.6% | 0.942 |
|  | Adult | 14.5% |  | 26.7% |  | 62.7% |  |
| **HIV status** | HIV negative | 8.1% | **<0.001** | 23.7% | **<0.001** | 70.1% | **<0.001** |
|  | HIV positive | 17.5% |  | 29.8% |  | 57.9% |  |
| **Category of TB** | New | 15.2% | 0.988 | 24.9% | **<0.001** | 63.7% | **<0.001** |
|  | Recurrent | 15.2% |  | 29.8% |  | 59.6% |  |
| **Mode of diagnosis** | Bacteriological | 14.7% | **<0.001** | 27.0% | **<0.001** | 62.3% | 0.092 |
|  | Clinical | 16.6% |  | 23.7% |  | 63.6% |  |

* p < 0.05 in bold font; COVID-19, Coronavirus disease of 2019; DS-TB, Drug- susceptible tuberculosis; HIV, Human immunodeficiency virus; ILTFU, Initial loss to follow-up; PTL, Post-treatment loss; TB, Tuberculosis.
