# Supplementary material for: A decline in tuberculosis diagnosis, treatment initiation and success during the COVID-19 pandemic, using routine health data in Cape Town, South Africa
Source: PLoS One. 2024 Sep 11;19(9):e0310383. doi: 10.1371/journal.pone.0310383 (PMC11389921; doi:10.1371/journal.pone.0310383)
Supplement: Attachment — Submitted filename: Response to reviewers PLOS ONE final clean.docx [file pone.0310383.s003.docx]

**2 August 2024**

**PLOS ONE**

**Manuscript Number:** PONE-D-24-18062

**Manuscript Title:** A decline in tuberculosis diagnosis, treatment initiation and success during the COVID-19 pandemic, using routine health data in Cape Town, South Africa

Dear Editor

Thank you for the opportunity to revise our manuscript and respond to the comments of the reviewers.

We have responded to each comment below in red and added the text from our revised manuscript in italics for ease of review. We appreciate the general supportive comments from the reviewer on the relevance of the work, the overall structure and analysis.

**Response to academic editor review**

1. Please ensure that your manuscript meets PLOS ONE's style requirements, including those for file naming. The PLOS ONE style templates can be found at

<https://journals.plos.org/plosone/s/file?id=wjVg/PLOSOne_formatting_sample_main_body.pdf> and

<https://journals.plos.org/plosone/s/file?id=ba62/PLOSOne_formatting_sample_title_authors_affiliations.pdf>

Thank you for this feedback. We have revised the format and changes are marked in track changes mode in the revised manuscript. Figure 1 had been uploaded as docx and has now been uploaded as a tiff file. The abstract is now under 300 words (previously 350).

2. Thank you for stating the following financial disclosure: 
 [This publication was supported by the South African Department of Science and Innovation (DSI) and the South African Medical Research Council (SAMRC) under the BRICS JAF #2020/101. Prof. Anneke C. Hesseling received the award. The content and findings reported herein are the sole deduction, view and responsibility of the researcher/s and do not reflect the official position and sentiments of the funders.<https://www.samrc.ac.za/>].  
Please state what role the funders took in the study.  If the funders had no role, please state: "The funders had no role in study design, data collection and analysis, decision to publish, or preparation of the manuscript." 
If this statement is not correct you must amend it as needed. 
Please include this amended Role of Funder statement in your cover letter; we will change the online submission form on your behalf.

Thank you for this feedback. It is correct that “The funders had no role in study design, data collection and analysis, decision to publish, or preparation of the manuscript."  Therefore please do change the online submission form accordingly on our behalf.

3. In the online submission form you indicate that your data is not available for proprietary reasons and have provided a contact point for accessing this data. Please note that your current contact point is a co-author on this manuscript. According to our Data Policy, the contact point must not be an author on the manuscript and must be an institutional contact, ideally not an individual. Please revise your data statement to a non-author institutional point of contact, such as a data access or ethics committee, and send this to us via return email. Please also include contact information for the third party organization, and please include the full citation of where the data can be found.

Thank you for this feedback. The data statement has been revised to refer to a non-author institutional point of contact (Western Cape Government Health & Wellness: Strategy & Health Support), with email address (Health.Research@westerncape.gov.za ) and process accessible via this URL: (https://www.westerncape.gov.za/general-publication/provincial-health-data-centre ). Reference #23 in the manuscript provides details of the Provincial Health Data Centre (Boulle A, Heekes A, Tiffin N, Smith M, Mutemaringa T, Zinyakatira, N, Phelanyane F, et al. Data Centre Profile: The Provincial Health Data Centre of the Western Cape. Int J Popul Data Sci. 2019;0(October).)

4. We notice that your supplementary figures are uploaded with the file type 'Figure'. Please amend the file type to 'Supporting Information'. Please ensure that each Supporting Information file has a legend listed in the manuscript after the references list.

Thank you for this feedback. The uploaded Figure 1 was intended to be included in the main body of the manuscript, not as supplementary material, hence uploaded as file type “Figure”. Reviewer #1 comment supports inclusion of Figure 1 in the main body of the manuscript.

5. Review Comments to the Author

Is the manuscript technically sound, and do the data support the conclusions?
The manuscript must describe a technically sound piece of scientific research with data that supports the conclusions. Experiments must have been conducted rigorously, with appropriate controls, replication, and sample sizes. The conclusions must be drawn appropriately based on the data presented. Have the authors made all data underlying the findings in their manuscript fully available?
Reviewer #1: Partly

The [PLOS Data policy](http://www.plosone.org/static/policies.action#sharing) requires authors to make all data underlying the findings described in their manuscript fully available without restriction, with rare exception (please refer to the Data Availability Statement in the manuscript PDF file). The data should be provided as part of the manuscript or its supporting information, or deposited to a public repository. For example, in addition to summary statistics, the data points behind means, medians and variance measures should be available. If there are restrictions on publicly sharing data—e.g. participant privacy or use of data from a third party—those must be specified

Reviewer #1: No (see details below)

There is a lag period of 5 months between the pre and during covid (October 2019 to March 2020). Five months is almost half the time period of study used by the authors and lot of changes can take place. The authors could provide a brief explanation as to why this time period was omitted.
Thank you for this comment. We used these periods to distinctly separate the periods. People started on treatment for TB between Oct 2019 and March 2020 would have been diagnosed with TB in the pre-COVID period but received treatment and outcomes recorded during the COVID period (as TB outcomes are recorded 6 months after starting TB treatment). We used a pre-COVID cohort referring to people diagnosed and having an outcome before COVID. The last time point that this was possible was September 2019.

This information was under Data collection in lines 145-148 ; “Pre-COVID-19 reflected the most recent period possible while including sufficient time to record the 6-month TB treatment outcomes. The during-COVID-19 period was pragmatically defined to start from 1 April 2020, following the onset of the first lockdown on 26 March 2020 (24)”

To make this easier to follow in the flow of the manuscript we have updated the study design section by moving this explanation there for clarity so that it now reads:

*Lines 112-117: This was an observational study comparing two retrospective annual DS-TB cohorts; pre-COVID-19 (1 October 2018 to 30 September 2019) and during-COVID-19 (1 April 2020 to 31 March 2021) cohorts. Pre-COVID-19 reflected the most recent period possible while including sufficient time to record the 6-month TB treatment outcomes. The during-COVID-19 period was pragmatically defined to start from 1 April 2020, following the onset of the first lockdown on 26 March 2020 (24).*

Methods
Lines 125 to 127 are part of the results and should not appear under methods.

Thank you for this comment. Lines 125 and 127 in the original manuscript (lines 133- 135 according to the Track changes updated version of the manuscript) are part of the Setting section and are not results, but background information about TB and HIV in CapeTown, with citations 20, 21, 22 and 23. (In 2019, there were 29,408 DS and 1,509 drug resistant (DR) TB diagnoses (20) in Cape Town. DS-TB treatment success was 76.3% in the 2018 cohort (21). Antenatal HIV prevalence was 22% (22) with 54% ART coverage (19).)

As this information appears under Setting, with relevant citations, we prefer to retain it as such.

The definition of terms provided is a necessary inclusion.

Thank you for this comment. We trust the definitions provided in Table 1, (lines 152- 154 according to the Track changes updated version of the manuscript), cover this sufficiently.

Results
Figure 1 should be included in the body of the manuscript as this is major results that answers the research question of comparing the two periods.

Thank you for this comment. We agree that Figure 1 should be included in the body of the manuscript. It is uploaded as a separate image file to comply with PLOS ONE submission requirements.

There are no table of results provided showing during Covid-19 period like table 3 and 4 for pre-covid 19. If there are too many then appendix should be provided.
Thank you for this comment and we agree that this information should be provided. We have included as supplementary material Tables S1 and S2, entitled:

“Table S1. DS-TB diagnosed, treated, and treatment success in the during-COVID-19 period (April 2020 to March 2021), in Cape Town, South Africa, disaggregated by demographic and clinical characteristics.”

“Table S2. Comparing initial loss to follow up (ILTFU), post-treatment loss (PTL) and cascade success for all individuals diagnosed with DS-TB in the during-COVID-19 period (April 2020 to March 2021) in Cape Town, South Africa, disaggregated by demographic and clinical characteristics.”

We have updated lines 222- 223 to refer to S1 and S2 Tables.

Lines 239-241: The authors are describing results that have not been presented, as there are no results for the covid-19 period.

Thank you for this comment, highlighting the importance of the inclusion of these data in Tables (it had been less explicitly included in Figure 1). We have included Tables S1 and S2 as supplementary material and updated lines 222-223 to refer to Tables S1 and S2. In addition, we have updated Figure 1 to show the treatment and cascade success rates and refer to Figure 1 in line 253.
